# Intense Sperm-Mediated Sexual Conflict Promotes Reproductive Isolation in *Caenorhabditis* Nematodes

**DOI:** 10.1371/journal.pbio.1001915

**Published:** 2014-07-29

**Authors:** Janice J. Ting, Gavin C. Woodruff, Gemma Leung, Na-Ra Shin, Asher D. Cutter, Eric S. Haag

**Affiliations:** 1 Department of Ecology and Evolutionary Biology, University of Toronto, Toronto, Ontario, Canada; 2 Department of Biology, University of Maryland, College Park, Maryland, United States of America; Cornell University, United States of America

## Abstract

Conflict between the sexes over reproductive interests can drive rapid evolution of reproductive traits and promote speciation. Here we show that inter-species mating between *Caenorhabditis* nematodes sterilizes maternal individuals. The principal effectors of male-induced harm are sperm cells, which induce sterility and shorten lifespan by displacing conspecific sperm, invading the ovary, and sometimes breaching the gonad to infiltrate other tissues. This sperm-mediated harm is pervasive across species, but idiosyncrasies in its magnitude implicate both independent histories of sexually antagonistic coevolution within species and differences in reproductive mode (self-fertilizing hermaphrodites versus females) in determining its severity. Consistent with this conclusion, in androdioecious species the hermaphrodites are more vulnerable, the males more benign, or both. Patterns of assortative mating and a low incidence of invasive sperm occurring with conspecific mating are indicative of ongoing intra-specific sexual conflict that results in inter-species reproductive incompatibility.

## Abstract 2

Sperm from other species invade female tissues to cause sterility and death, helping to keep nematode species boundaries intact.

## Author Summary

The sexes have divergent reproductive interests, and conflict arising from this disparity can drive the rapid evolution of reproductive traits and promote speciation. Here we describe a unique reproductive barrier in *Caenorhabditis* nematodes that is induced by sperm. We found that mating between species can sterilize maternal worms and even cause premature death, and we were able to attribute this phenomenon directly to the sperm themselves. Sperm from other species can displace sperm from the same species and, in some cases, can invade inappropriate parts of the maternal reproductive system and even their non-reproductive tissues. We find that mating to males of another species harms females far more than does within-species mating. Overall, our observations are consistent with ongoing sexual conflict between the sexes within species, arising as a byproduct of sperm competition among the gametes of different males. Finally, patterns of assortative mating indicate that mating behaviours that reduce the likelihood of costly inter-species mating have evolved in this group of animals. These findings support an important role of sexual selection and gametic interactions contributing to reproductive boundaries between species, as predicted by evolutionary theory.

## Introduction

Rarely do reproductive interests of males and females perfectly align. Sexual selection can accelerate the evolution of the traits and molecules mediating reproductive encounters, and this can lead to sexual conflict [1],[2]. Components of the reproductive system that mediate male-female interactions, such as reproductive tract morphology, sperm and egg traits, and molecular components of seminal fluid all diverge rapidly between many species [3],[4]. The particularly forceful process of sexual antagonism drives co-evolutionary arms races between sex-limited traits that exact or counteract harmful, but self-serving, effects on the other sex [2],[4],[5]. Ongoing sexually antagonistic coevolution that operates within a species might generate mismatched interactions between gametes or other reproductive tract components when mating occurs between species. When such mismatches interfere with normal conspecific reproduction [6],[7], they have the potential to instigate or magnify reproductive isolation between species [8],[9]. Selection for traits that prevent the deleterious consequences of inter-species mating for the parents or hybrid offspring may result in further trait evolution [10]. Though pre-mating reinforcement behaviours have received much attention and debate [10]–[12], post-mating mechanisms of gametic isolation, such as conspecific sperm precedence, also can play key roles in pre-zygotic reproductive isolation [13],[14].

The prevailing view of gametic isolation between species is that fertilization precedence of conspecific sperm can provide a potent reproductive barrier, mediated by cryptic female choice, sperm competition, or incompatibilities between female reproductive tracts and heterospecific ejaculates [15]–[18]. Conspecific sperm precedence occurs both in species with internal and external fertilization, governed by a broad variety of proximate mechanisms [14],[19],[20].

Alternatively, *Drosophila* provide examples of inter-species mating harm, for example, owing to an overly engorged “insemination reaction mass” that exacts a fitness cost on females [6],[7], and female *Carabus* beetles suffer ruptured reproductive tracts from physical damage upon inter-species matings [21]. Within species, male seminal proteins can manipulate female physiology in a manner sub-optimal for females but beneficial to males [22]. While coevolution between the sexes may obscure the traces of such sexual antagonism (as for other forms of genetic conflict [23]), interactions between divergent populations and species can unmask the underlying conflicts by revealing mismatched male and female traits [8].

*Caenorhabditis* nematodes provide a powerful system to examine both sexual antagonism and its modulation by reproductive mode. Males, females, and hermaphrodites will mate readily and promiscuously in lab culture, and mechanical harm incurred from multiple mating reduces longevity and survival in *C. elegans* hermaphrodites [24] and *C. remanei* females [25]. Male-derived chemical cues also are thought to accelerate female and hermaphrodite aging [26]. Following copulation, *C. elegans* hermaphrodites can expel male ejaculates [27], and males deposit copulatory plugs that inhibit re-mating [28],[29] and induce larger brood sizes in their partners [30]. In response to experimentally elevated sperm competition, *C. elegans* evolve larger sperm [31]. Though anatomical evolution in *Caenorhabditis* is conservative, these intra-specific dynamics suggest there may be substantial inter-species divergence in cryptic reproductive traits.

Evolutionary transitions in reproductive mode from highly outbreeding to highly self-fertilizing are expected to reduce intra- and inter-sexual conflict [32],[33]. Three species of *Caenorhabditis* have independently evolved androdioecy (hermaphrodites and males) from dioecy (females and males), such that hermaphrodites are capable of self-fertilization in addition to being fertilized by males [34]. These androdioecious species manifest a “selfing syndrome” analogous to plants that includes reduced sperm size and low mating vigor [35],[36]. Hermaphrodites from such species with relaxed sexual selection might be particularly susceptible to adverse effects of mating to vigorous males from closely related species that have a recent history of strong sexual selection (the “weak inbreeder, strong outbreeder” or WISO hypothesis [33],[37]). Despite the generally limited understanding of *Caenorhabditis* ecology and inter-species interactions in their rotting fruit and vegetal habitats, some species are sympatric [34],[38], putting them at risk for inter-species encounters. Species readily mate with one another in the laboratory, and the animals' transparent bodies provide literal windows into postmating-prezygotic, and postzygotic, reproductive interactions and barriers [39]–[41].

Here we describe an unprecedented postmating-prezygotic reproductive barrier in *Caenorhabditis* nematodes, induced directly by sperm cells, that imposes potent fitness costs to females and hermaphrodites. Theory predicts species with selfing hermaphrodites to be more susceptible to inter-species harm and less capable of inducing harm [33],[37]. Theory also predicts that rapid divergence in sexually selected traits will produce heterogeneity in harmful effects between species pairs and consequently may fail to yield phylogenetic signal in the magnitudes of effect [2],[4]. In this counterpoint to mechanisms of conspecific sperm precedence, we affirm a potent role for sexual conflict as a pre-zygotic isolating barrier between species.

## Results

### Mating to Males of a Different Species Is Harmful

**Figure 1:**
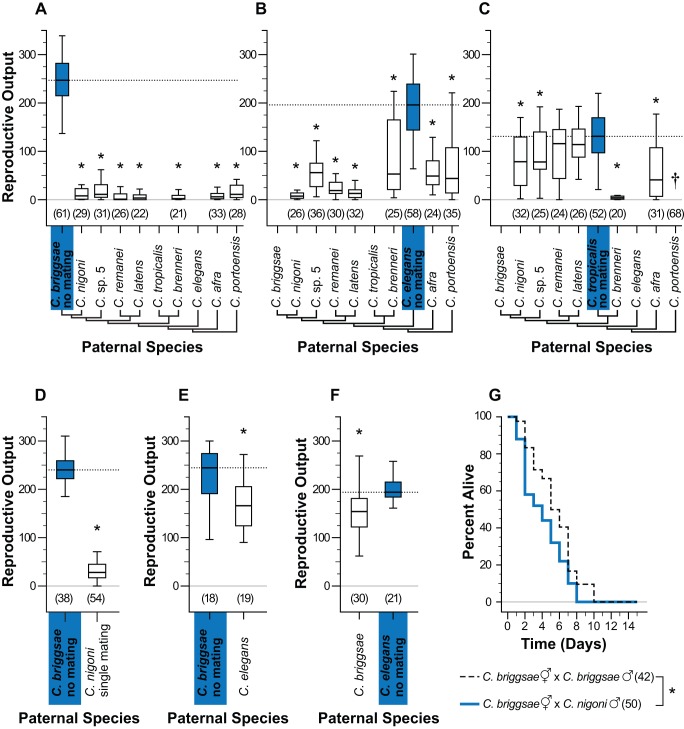
Impacts of inter-species matings on hermaphrodites. Hermaphrodites of each selfing species were mated to males of dioecious (A–C) or another androdioecious (D, E) species and reproductive output was quantified as the number of self-progeny produced two days post mating treatment. (A) *C. briggsae* and (B) *C. elegans* produced significantly fewer progeny after heterospecific matings to males of all species tested (white) compared to selfing or “no mating” (blue). (C) *C. tropicalis* also produced fewer progeny after heterospecific matings, but only to a subset of the species tested. (D) A single heterospecific mating event was sufficient to induce progeny loss for *C. briggsae* hermaphrodites crossed to *C. nigoni* males (Mann-Whitney U = 37.0, *p*≤0.001). (E, F) Males from hermaphroditic species also reduced hermaphrodite self-progeny production following heterospecific mating (*C. briggsae* U = 65.0, *p* = 0.001; *C. elegans* U = 178.0, *p* = 0.008). However, males from hermaphroditic species were less effective at reducing progeny compared to dioecious males (*C. briggsae* U = 122.0, *p*≤0.001; *C. elegans* U = 631.0, *p*≤0.001). (G) *C. briggsae* hermaphrodites mated to *C. nigoni* males (solid blue line) suffer higher mortality than when mated to *C. briggsae* males (dashed black line; Kaplan-Meier log-rank test: χ^2^ = 5.218, df = 1, *p* = 0.022). Asterisks in (A–F) indicate significant difference from selfing control (Mann-Whitney U test *p*<0.05; Bonferroni correction applied in (A–C); (A, C) corrected α = 0.007; (B) corrected α = 0.008; Table S1). In (A–F), boxplot whiskers indicate 1.5× (interquartile range) and dotted horizontal lines indicate median of viable progeny produced by the selfing control for reference. Dagger (†) in (C) indicates that no successful matings occurred between *C. portoensis* males and *C. tropicalis* hermaphrodites. Sample sizes are shown in parentheses. Phylogeny in (A–C) adapted from [34].

In no-choice mating arenas, *Caenorhabditis* copulate repeatedly (Figure S1; Text S1), and will readily mate with different species [41]–[43]. We first examined the impact of mating the self-fertile hermaphrodites of *C. briggsae*, *C. elegans*, and *C. tropicalis* [44] with males of other species (Figure 1A–1C; Text S1). Despite the presence of abundant self-sperm, offspring production was invariably compromised. By contrast, mating to conspecific males leads to increased reproductive output, because hermaphrodite sperm stores are supplemented by the male sperm (Figure S2) [45]. The extremity of reduced reproductive output upon inter-species mating, however, depends in part on the identity of maternal and paternal partners. For example, *C. briggsae* and *C. elegans* hermaphrodites produced <5% and <30% of their normal brood, respectively, when mated with any of seven dioecious species of *Caenorhabditis* tested (Figure 1A and 1B). *C. tropicalis* hermaphrodites, however, showed similarly striking sensitivity to males of *C. brenneri*, but were resistant or less sensitive to males of other species (Figure 1C).

In crosses between *C. briggsae* hermaphrodites and *C. nigoni* males, a single heterospecific mating event is sufficient to strongly depress reproductive output (Figure 1D), and prolonged exposure accelerates hermaphrodite mortality beyond that seen in conspecific or reciprocal crosses (Figure 1G). Increasing the abundance of conspecific sperm in hermaphrodites by mating them with conspecific males immediately before or after the heterospecific matings was insufficient to prevent or rescue sterilization of *C. briggsae* hermaphrodites by *C. nigoni* males (Figure S2). Males from androdioecious species of both *C. elegans* and *C. briggsae* were markedly less able to reduce the reproductive output of heterospecific hermaphrodites compared to the effect of dioecious males (Figure 1E and 1F). However, there is no clear association between degree of sterilization and phylogenetic distance (Figure S3A), although notably *C. tropicalis* is most sensitive to males of its closest tested relative, *C. brenneri*.

The above observations demonstrate that (i) hermaphrodites of all three self-fertile species are susceptible to adverse effects of mating with males of at least some other species and (ii) males of all species are capable of adversely affecting the reproductive output of hermaphrodites of at least some species. Such disparities among species in gametic isolation are predicted to be a common outcome of sexual selection on sperm competition [37]. Consistent with intense intra-species sperm competition, multiple mating is readily observed in laboratory populations of outbreeding species (Figure S1; Text S1), while the rarity of males in self-fertile populations [46],[47] suggests that multiple mating in these lineages is rare. Additionally, males from highly selfing species are less harmful to heterospecific mates, consistent with weak inbreeder, strong outbreeder (WISO) dynamics [33],[37].

**Figure 2:**
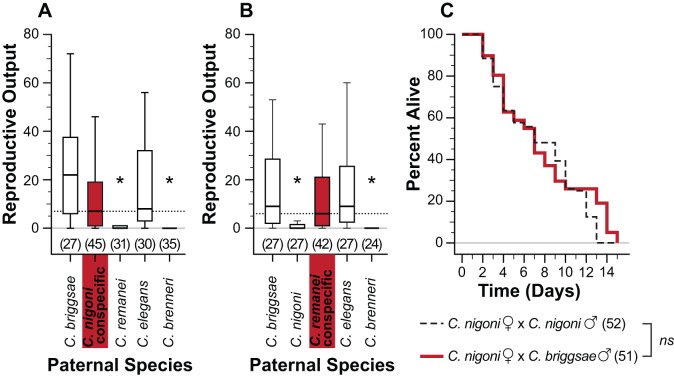
Impacts of inter-species matings on females. Females from dioecious species (A: *C. nigoni*, B: *C. remanei*) were initially mated overnight to conspecific males, followed by overnight mating of treatment females (white) to heterospecific males from either dioecious (*C. nigoni, C. remanei, C. brenneri*) or androdioecious (*C. briggsae*, *C. elegans*) species; control females (red) did not receive a second mating. Reproductive output was then quantified as the number of progeny produced two days post mating treatment. (A, B) Females often became sterilized upon a subsequent mating with heterospecific males from dioecious species compared to controls: (A) *C. nigoni* females (*C. remanei* males U = 385.5, *p* = 0.001; *C. brenneri* males U = 206.5, *p*≤0.001), (B) *C. remanei* females (*C. nigoni* males U = 239.5, *p*≤0.001; *C. brenneri* males U = 259.5, *p*≤0.001). However, females were resistant to harm induced by males of androdioecious species: (A) *C. nigoni* (*C. briggsae* males U = 409.5, *p* = 0.020; *C. elegans* males U = 577.0, *p* = 0.291), (B) *C. remanei* (*C. briggsae* males U = 887.0, *p* = 0.886; *C. elegans* males U = 863.0, *p* = 0.731). (C) *C. nigoni* females mated to heterospecific *C. briggsae* males (solid red line) did not incur a statistically significant survival cost relative to conspecific mating (dashed black line; Kaplan-Meier log-rank test: χ^2^ = 0.203, df = 1, *p* = 0.652; *ns* not significant). Asterisks in (A and B) indicate significant difference from controls (Bonferroni correction for multiple tests were applied; corrected α = 0.0125). Boxplot whiskers in (A and B) indicate 1.5× (interquartile range) and dotted horizontal lines indicate median of viable progeny produced by the controls (females mated with conspecifics); sample sizes are shown in parentheses.

To assess the generality of heterospecific sterilization, we also examined male effects on females of dioecious species (Figure 2). Despite their similar sperm sizes [48],[49], heterospecific mating of dioecious males from *C. remanei*, *C. nigoni*, and *C. brenneri* reduced the reproductive output of females of *C. remanei* and *C. nigoni* (Figure 2A and 2B). However, males from highly selfing species lacked the capacity to compromise the fecundity of heterospecific females (Figure 2A and 2B).

In *Drosophila*, different levels of sexual antagonism can lead to reduced female survivorship in inter-strain matings [50]. To explore whether heterospecific *Caenorhabditis* matings exert negative effects beyond reproductive output, we quantified maternal survival in the four cross combinations involving *C. briggsae* and its outbreeding sister species, *C. nigoni*. Mating with *C. nigoni* males significantly reduced the lifespan of *C. briggsae* hermaphrodites relative to conspecific mating (Figure 1G), but did not adversely affect conspecific *C. nigoni* female survival in our assay (Figure 2C), consistent with additional harmful effects of heterospecific mating beyond sterilization. Matings between another closely related selfer-outbreeder pair produced a different pattern, in which males from the selfing *C. tropicalis* exerted a much weaker effect on *C. wallacei* female longevity than did the other three cross combinations (Figure S4A). Matings between dioecious species yielded heterogeneous impacts on maternal survival. *C. nigoni* and *C.* sp. 5 males produced no significant reduction in female survival beyond that seen for conspecific matings (Figure S4B), whereas *C. nigoni* asymmetrically harmed *C. remanei* survival (Figure S4C). Similar to sterilization, then, inter-species mating affects maternal survival with species-pair dependencies in both outbreeding and selfing species. This observation is consistent with distinct evolutionary resolutions of sexual conflicts in different lineages. Additionally, we see a compelling effect of reproductive mode: in selfing species, hermaphrodites are more vulnerable, the males more benign, or both (as for *C. briggsae*).

**Figure 3:**
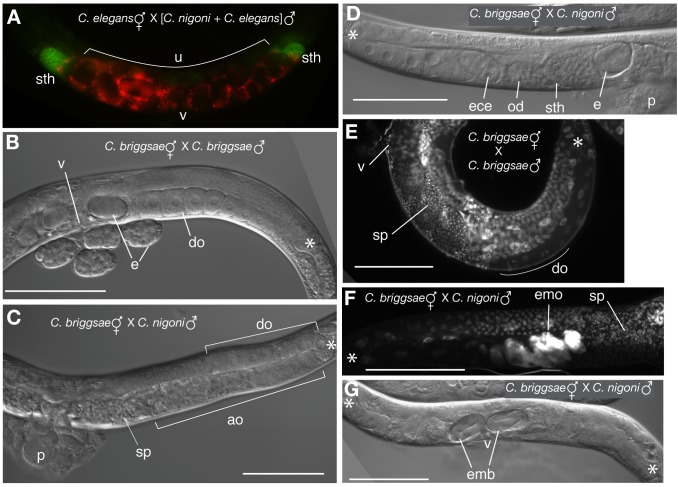
Mechanisms of sterilization by heterospecific males. (A) Shortly after mating, the smaller *C. elegans* male sperm (red) are displaced from the spermatheca by larger *C. nigoni* sperm (green) in *C. elegans fog-2* “females” doubly mated to vitally dyed males. sth, spermatheca; u, uterus, v, vulva. This panel is a mosaic assembled from multiple overlapping images. (B) *C. briggsae* adult hermaphrodite after overnight mating with conspecific males. Abundant embryos (e) are restricted to the uterus and reliably laid through the vulva (v). Diakinesis stage oocytes (do) are seen distal to the uterus, but not beyond the bend in the reflexed gonad (asterisk). (C) When mated overnight with *C. nigoni* males, few embryos are produced despite abundant sperm (sp). Proximal oocytes are abnormal (ao), and diakinesis oocytes are pushed distal to the gonad bend. p, copulatory plug. (D) In hermaphrodites recently inseminated by heterospecific males, the germ line is generally well organized, but ectopic embryos (ece) can be observed distal to the oviduct (od), which is often distended by sperm that have breached it from the spermatheca (sth). Embryos are also seen in the normal uterine position, explaining how some cross progeny are laid [39]. (E) Hoechst DNA staining of conspecifically mated *C. briggsae* reveals abundant punctate sperm nuclei (sp) that are always restricted to the uterus and spermatheca and multiple diakinesis-stage meiotic nuclei (do). (F) DNA staining of *C. briggsae* hermaphrodites mated overnight with *C. nigoni* (similar to B) reveal a zone of endomitotic oocytes (emo) in the proximal region of the ovary abutting a large mass of sperm (sp). (G) In some cases, embryos localized to the uterus (emb) are viable (as judged by their advanced state of development) but not laid through the vulva (v).

One possible mechanism of reduced female fitness is competitive displacement of conspecific sperm by heterospecific sperm. Hermaphrodite *Caenorhabditis* make relatively small sperm that compete poorly with even conspecific male sperm, and their conspecific males make sperm that are smaller than those of dioecious species' males [31],[48],[49],[51]. Thus, the sterilization of hermaphrodites could result from the displacement of self-sperm by larger, yet ineffectual, heterospecific sperm [41]. To address this issue, we labeled *C. elegans* and *C. nigoni* males with vital dyes, mated them to phenotypically female *C. elegans fog-2* animals, and observed the transferred sperm from each male in live animals. Indeed, within a few hours of mating, we observed strong displacement of the smaller *C. elegans* male sperm from the spermathecae by the larger *C. nigoni* sperm (Figures 3A and S6C). However, sperm displacement does not explain the adverse effects of heterospecific mating on survivorship nor does it account for the seeming irreversibility of sterility induced by males upon their mating partners.

### Invasive Sperm Are the Primary Cause of Male Harm

*C. briggsae* hermaphrodites mated to *C. nigoni* males display striking germ line abnormalities, including disorganized proximal germ cells and ectopic, distally localized diakinesis-stage oocytes (Figure 3C). In addition, embryos often formed distal to the oviduct (Figure 3D), and, more rarely, we observed egg-laying defects (Figures 3G and S5). The proximal mass of disorganized germ cells is reminiscent of ovulation-defective mutant phenotypes in *C. elegans* [45],[52],[53]. Consistent with this, DNA staining of *C. briggsae* hermaphrodites revealed extensive endomitotic oocyte accumulation after one day of mating with *C. nigoni* males (54%, *n* = 78; Figure 3F), which increased further after a second day (91%, *n* = 70). No endomitotic oocytes were observed among *C. briggsae* hermaphrodites mated to conspecific males (*n* = 51; Figure 3B), indicating that *C. nigoni* males promote oocyte maturation defects in *C. briggsae* hermaphrodites.

**Figure 4:**
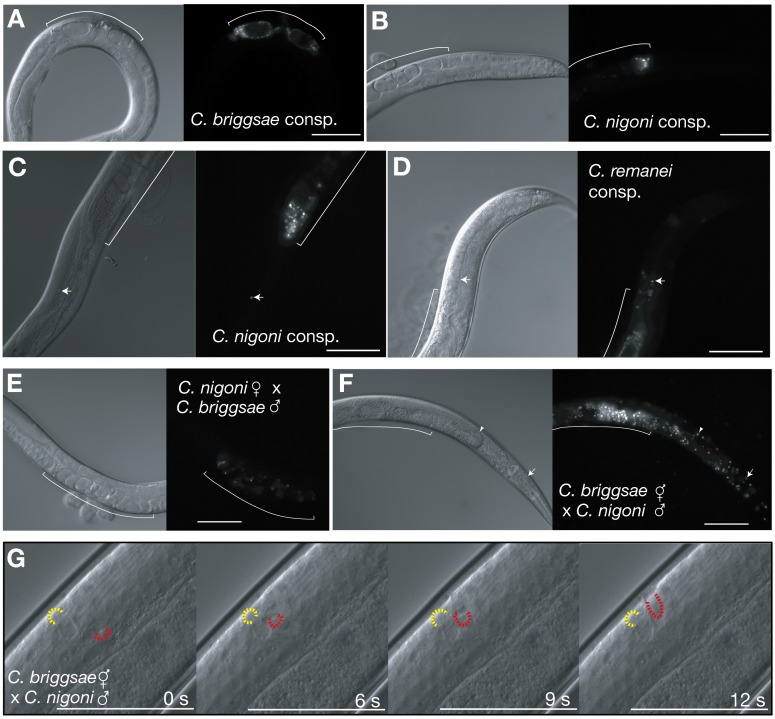
Heterospecific sperm invade maternal tissues. (A–F) Paired images of DIC and vitally dyed sperm inside maternal animals after conspecific and heterospecific crosses. (A) In conspecific crosses with *C. briggsae*, sperm remain completely in the uterus and spermatheca (bracket). (B) Female from *C. nigoni* conspecific cross displaying sperm localization to the uterus and spermatheca. (C and D) In rare cases of conspecific crosses for *C. nigoni* (C; 5%) and *C. remanei* (D; 8%), we observe isolated sperm (arrows) breach the distal spermathecal valve, but this does not cause sterility. (F) When *C. nigoni* males are mated with *C. briggsae* hermaphrodites, ectopic sperm are seen in large numbers, and in some cases breach the gonad completely. Wedge, anterior end of ovary; arrowhead, sperm nuclei in head region. (G) Series of frames from time-lapse movie (Movie S2) depicting movement of an individual ectopic *C. nigoni* sperm in the distal gonad of a *C. briggsae* hermaphrodite. The yellow crescent outlines the cell body of a sessile sperm, which serves as a reference point to highlight the motion of a second sperm, whose body is outlined in red.

Males transfer both sperm and seminal fluid components to mates, and in principle, either might negatively affect their partner's reproduction and lifespan after heterospecific matings. To distinguish these possible mechanisms, we again applied a fluorescent vital dye to males, mated them to conspecific or heterospecific individuals, and observed transferred sperm in live animals. After six hours of mating to *C. briggsae* hermaphrodites, conspecific male sperm had localized to the spermathecae and uterus in all animals (*n* = 52), as expected (Figure 4A; Movie S1). In a striking contrast, we found that 90% of *C. briggsae* hermaphrodites mated heterospecifically to *C. nigoni* males had sperm present in ectopic locations in the distal and proximal gonad, whereas only 10% of animals had sperm properly localized exclusively to the spermathecae or uterus (*n* = 188; Figures 4F and S6A; Movies S1 and S2). This appears to begin when many sperm penetrate the distal spermathecal valve, which normally separates maturing oocytes from sperm. Further, 7% of the hermaphrodites showed invasion of sperm into the body cavity (*n* = 159; Figure 4F; see Movie S1).

Sperm of *C. nigoni* males also commonly invaded the gonad past the spermatheca when mated to other species (*C. elegans* and *C. remanei*; Figures S5, S6A–S6C), but the effect was most extreme in *C. briggsae* hermaphrodites, consistent with its pronounced deficits in reproductive output. Just three hours after mating, more than 50% (*n* = 72) of *C. briggsae* hermaphrodites had *C. nigoni* sperm in the distal gonad (cf. 90% six hours post mating; Figure S6A and S6D), whereas ∼20% (*n* = 39) of *C. elegans* and 2.9% (*n* = 35) of *C. tropicalis* hermaphrodites were similarly afflicted (Figure S6B). Ectopic sperm also were very rarely observed in *C. tropicalis* hermaphrodites mated heterospecifically to *C. brenneri* and *C. remanei* (0.9%, *n* = 111 and 0%, *n* = 18, respectively; Figure S6B), consistent with the infrequency of sterilization observed for this species. The ability of *C. nigoni* sperm to overrun the spermatheca is greater than that of other species tested. *C. remanei* sperm were rarely found in ectopic locations in *C. nigoni* females (5%, *n* = 100; Figure S6A), and ectopic sperm originating from *C. briggsae* males were never observed (*n* = 70; Figure 4A and 4E). Notably, the distinctly stronger sterilization of *C. tropicalis* by males of *C. brenneri* than *C. nigoni* occurs in the face of similar sperm localization patterns three hours post-mating (Figures 1B and S6C), further supporting the idea that factors other than sperm displacement are important in heterospecific sterilization. Intriguingly, in conspecific matings with *C. nigoni* and *C. remanei*, 5%–8% of females had small numbers of sperm in ectopic gonad locations (Figures 4C, 4D, and S6A). This low but detectable incidence of mislocalized sperm in conspecific matings is indicative of ongoing sperm-driven sexual conflict within dioecious species.

**Figure 5:**
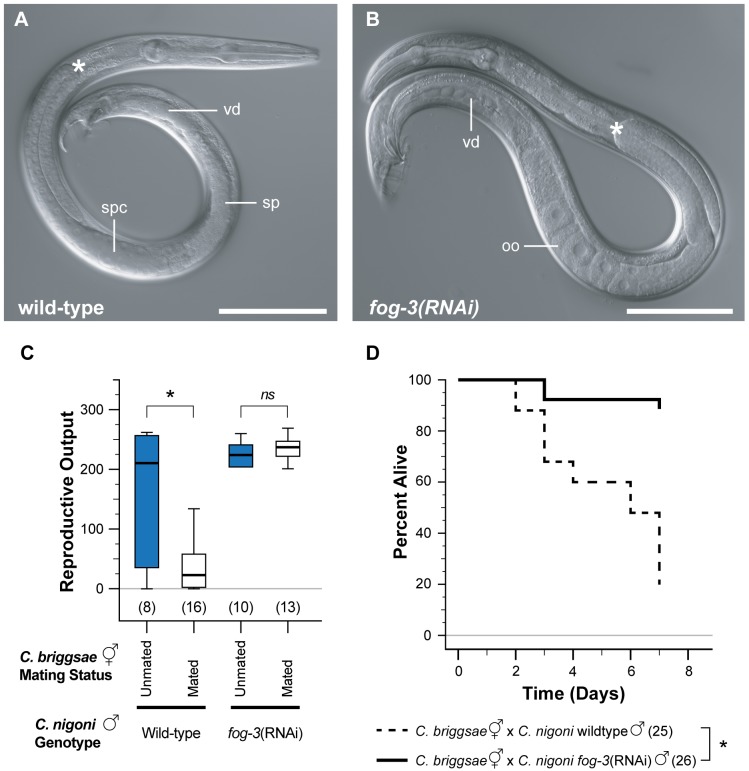
Sperm cause sterility and early mortality in cross-species matings. (A) Wild-type *C. nigoni* male, with spermatocytes (spc) and sperm (sp) in the seminal vesicle. These are extruded through the vas deferens (vd) during copulation. (B) *C. nigoni* male treated with maternal *Cni-fog-3(RNAi)*. Oocytes (o) develop instead of sperm, while the somatic testis, including the glandular vas deferens (vd), remain unaffected. (C) The number of viable progeny of *C. briggsae* hermaphrodites do not differ when unmated (blue) or mated (white) to germline-feminized (*fog-3 (RNAi)*) *C. nigoni* males (Mann-Whitney U = 51.5, *p* = 0.419; *ns*, not significant), unlike when unmated or mated to wild-type *C. nigoni* males (U = 26.5, *p* = 0.019). Boxplot whiskers indicate 1.5× (interquartile range). (D) *C. briggsae* hermaphrodites mated to *Cni-fog-3 (RNAi)* males (solid line) have significantly reduced mortality compared to when mated to wildtype *C. nigoni* males (dashed line; Kaplan-Meier log-rank test: χ^2^ = 23.7, df = 1, *p*≤0.001). Sample sizes are shown in parentheses.

Negative effects of seminal components contributed by somatic glands can potentially trigger harmful reactions in females and hermaphrodites [54]–[56]. We therefore tested for a direct role of sperm in causing harm to *C. briggsae* hermaphrodites by feminizing the germ line of *C. nigoni* males via *fog-3(RNAi)* (Figure 5A and 5B) [57]. Treated males have a normal somatic testis, mate normally, and can deposit seminal components including the copulatory plug, but transfer no sperm. The reproductive output (Figure 5C) and survival (Figure 5D) of *C. briggsae* hermaphrodites mated to spermless *C. nigoni fog-3(RNAi)* males is strikingly higher than that of animals mated to wild-type *C. nigoni* males. These observations implicate sperm infiltration into the gonad arms (and potentially into the body cavity) as the principal cause of male-induced permanent harm in heterospecific matings.

### Assortative Mating of Species

The extreme fitness costs to inter-species mating might select for behaviours that promote assortative mating [10],[12],[14],[19]. We first examined female avoidance of heterospecific males because *Caenorhabditis* males mate indiscriminately when given the opportunity, and mating pheromones produced by virgin females are strongly attractive to males across species [43],[58]; tests for assortative mating in choice experiments have not been reported previously between *Caenorhabditis* species. We created assay populations with an equal mixture of females from each of two species and males from one of them (reciprocally), and then quantified the incidence of avoidance behaviour and of mating success of each species, as evidenced by copulatory plugs placed onto the vulva. This assay design presumes that dioecious males mate indiscriminately [43] and thus that female and hermaphrodite behaviour will dominate mating outcomes (Text S1).

**Figure 6:**
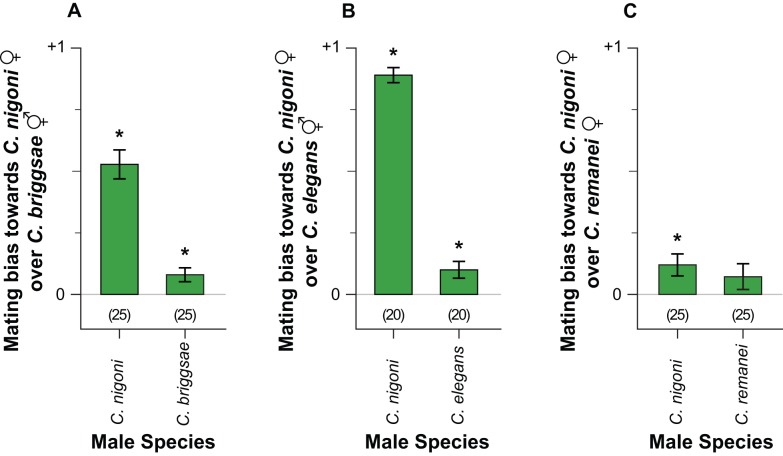
Assortative mating behavioural responses. In assay arenas containing a mixture of females from two species and males from one species, (A) *C. nigoni* females are more likely than *C. briggsae* hermaphrodites to mate with *C. briggsae* males (t = 2.828, df = 24, *p* = 0.009) and *C. nigoni* males (t = 8.988, df = 24, *p*≤0.001). (B) A similar female mating bias was also observed when *C. elegans* hermaphrodites and *C. nigoni* females were presented to *C. elegans* males (t = 2.939, df = 19, *p* = 0.008) and to *C. nigoni* males (t = 28.996, df = 19, *p*≤0.001). (C) Mixtures of *C. remanei* and *C. nigoni* females show a mating bias toward *C. nigoni* females over *C. remanei* females when they are presented with *C. nigoni* males (t = 2.683, df = 24, *p* = 0.013), but not when presented with *C. remanei* males (t = 1.365, df = 24, *p* = 0.185). Positive values indicate that *C. nigoni* females mated more readily than the other female (or hermaphrodite) species that they were paired with; negative values indicate the reciprocal, while zero indicate no mating bias. Error bars represent ±1 standard error (SE); sample sizes are shown in parentheses.

Owing to their aggressive sperm, we first focused on copulatory responses to *C. nigoni* males. Overall, we observed that *C. nigoni* females exhibit a conspecific mating bias (Figure 6A–6C). We introduced *C. nigoni* females and males to either *C. briggsae* hermaphrodites (Figure 6A), *C. elegans* hermaphrodites (Figure 6B), or *C. remanei* females (Figure 6C). *C. nigoni* males were favoured by conspecific females over all heterospecific mating partners. When *C. remanei* and *C. nigoni* females (Figure 6C) were presented with *C. remanei* males, the females of both species showed no mating biases and were equally likely to mate with the males. This contrasts with the conspecific mating bias we observed when these females were presented with *C. nigoni* males (Figure 6C). This discrepancy suggests stronger assortative mating bias in favour of conspecifics by *C. nigoni* females compared to *C. remanei* females.

We also tested *C. nigoni* females in the presence of either *C. brigggsae* hermaphrodites and males (Figure 6A) or *C. elegans* hermaphrodites and males (Figure 6B). Interestingly, *C. nigoni* females mated more readily with the heterospecific males than did the hermaphrodites to males of their own species (Figure 6A and 6B). The insensitivity of *C. nigoni* female reproductive output to having mated with androdioecious males (Figure 2A) could partially contribute to a lack of mate avoidance towards this class of heterospecific males (Figure 6A and 6B), although female sex pheromones [58] and hermaphrodite mating avoidance behaviours [43] also could contribute to this outcome. In addition to the mating outcomes, *C. briggsae* hermaphrodites were distinct in that they crawled away from the mating area when in the presence of *C. nigoni* males (t = −2.449, degree of freedom [df] = 44.672, *p* = 0.018). We interpret leaving the mating area as avoiding copulatory attempts by males.

## Discussion

**Figure 7:**
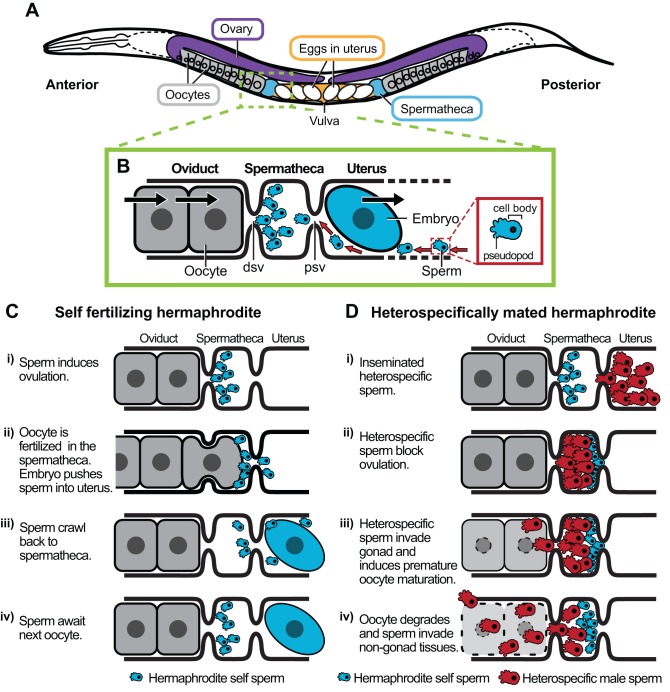
Graphical representation of a model for gametic isolation in *Caenorhabditis*. (A) Diagram of the reproductive tract of a XX nematode animal, magnified in (B) to illustrate the typical movement of gametes near the site of fertilization. Sperm must repeatedly crawl from the uterus to the spermatheca *via* the proximal spermathecal valve (psv) after being pushed out by fertilized eggs, migrating toward attractants secreted by the most proximal oocytes; red arrows indicate direction of sperm movement. Oocytes in the oviduct enter the spermatheca through the distal spermathecal valve (dsv) to be fertilized, then move into the uterus and exit the animal through the vulva; black arrows indicate direction of oocyte and embryo movement. (C) The normal process of self-fertilization in hermaphrodites (adapted from [64]) becomes disrupted upon heterospecific mating. (D) Heterospecific male sperm inhibit ovulation, invade the proximal gonad, induce premature oocyte maturation and ectopic fertilization, and in some cases invade somatic tissues.

Here we show that sperm transferred from matings between species of *Caenorhabditis* nematodes induce severe damage to females, compromising their reproduction and longevity. This phenomenon is the antithesis of the conspecific sperm precedence observed in diverse organisms, but still yields a form of gametic isolation owing to sperm displacement and ectopic sperm migration by heterospecific sperm. *Caenorhabditis* females commonly mate with multiple males (Figure S1; Text S1), and sperm competition selects for more aggressive sperm within species [31]. Attracted to oocyte-secreted chemical cues, sperm must repeatedly crawl to the spermatheca (the site of fertilization) as embryos push them into the uterus (Figure 7) [45],[59]. We propose that intra-specific sperm competition between males within the reproductive tracts of multiply mated females acts to select for aggressive sperm in an evolutionary intra-species arms race. Sperm migration into distal portions of the gonad would represent a byproduct of sperm competition with harmful consequences for females, that in turn leads to counter-selection for female resistance [4],[60],[61]. While co-evolution within a species largely keeps these interactions matched, male and female changes can fail to complement one another in an inter-species context. Although such divergence need not have precipitated the initial isolating events, they nevertheless contribute to extant reproductive isolation among species. It remains to be tested whether heterospecific sterilization by sperm could also be co-opted as a weapon in inter-species resource competition when multiple *Caenorhabditis* species inhabit the same resource patch.

This model of sperm competition (Figure 7D) and sexual conflict predicts sexual antagonism as an ongoing selective pressure within species of *Caenorhabditis*. We find evidence supporting this idea in the low incidence of ectopic sperm migration observed in conspecific matings for *C. remanei* and *C. nigoni* (Figure S6A). This model also could explain fertility patterns reported for crosses between parthenogenic and amphimictic *Aphelenchus* nematodes [62], suggesting applicability of this mode of gametic isolation across diverse taxa. Interactions between species with overlapping geographic ranges, as occurs for some *Caenorhabditis* including *C. briggsae* with *C. nigoni* [34] and *C. tropicalis* with several species [38], could select for behavioural or gametic strengthening of species boundaries or yield reproductive character displacement [10],[63], consistent with some patterns of assortative mating and sperm migration observed in our experiments (Figures 6, S6A, and S6B). However, further tests comparing sympatric and allopatric genotypes are needed [10],[14]. This model further predicts the evolution of distinct cellular and molecular mechanisms mediating male sperm vigor and female resistance in different species, and relaxation of selection in some highly self-fertilizing hermaphrodite species [33],[37]. Consistent with what we observe empirically, differences among species in the mechanisms of evolutionary response to intra-species sexual conflict will create phylogenetic heterogeneity in how strongly the inter-species mismatches manifest as harm to females and hermaphrodites.

We addressed several other potential mechanisms that could contribute to the compromised maternal fecundity and longevity upon inter-species mating. (i) Trauma through copulation may occur in *Caenorhabditis* [24], and repeated matings in *Drosophila melanogaster* can induce female sterility and mortality [54]. We can rule out this possibility because germline-feminized males with normal copulatory behaviours do not affect maternal reproductive output or longevity (Figure 5C and 5D). We also exclude repeated matings as an explanation for reduced reproduction because a single inter-species mating event also severely diminishes hermaphrodite reproductive output (Figure 1D). (ii) We can rule out insufficient sperm or oocytes as the cause of sterilization, as hermaphrodites cease laying embryos despite the abundance of oocytes and sperm in the reproductive tract (Figure 3) [40]. (iii) The reduction in progeny production after heterospecific mating could result from superior fertilization capability of heterospecific sperm, leading to mortality of hybrid embryos, as post-zygotic reproductive isolation is nearly complete between most species studied here. However, we and others have observed insufficient numbers of dead eggs to account for the overall reduction in reproductive output [40]–[42]. (iv) Larger sperm from males of another species also could potentially outcompete and displace the conspecific sperm from the spermatheca, yielding fewer progeny [64]. Indeed, such displacement occurs initially following heterospecific matings to males of a species with larger sperm (Figure 3A). However, hermaphrodite sperm for *C. briggsae*, *C. elegans*, and *C. tropicalis* all are similarly small [48],[49], and yet these species differ starkly in susceptibility to the negative effects of mating to heterospecific males (Figure 1A–1C). Likewise, the disparity in sperm size of males from different dioecious species does not correlate with the extremity of reduced progeny production (Figure S3), and would not be expected to shorten lifespan. These observations reinforce the direct effects of sperm mislocalization in the maternal reproductive tract as the primary mechanism of male-induced permanent harm in inter-species matings.

Although our experiments demonstrate the key role of sperm cells in heterospecific harm upon mating, non-sperm components also could contribute. For example, upon maturation, *C. elegans* sperm cells release by exocytosis the contents of their lysosome-like membranous organelles (MOs) [65], and it is conceivable that molecules from this sperm cell-derived component of the seminal fluid could interact with female tissues. Indeed, the nematode major sperm proteins (MSPs) act as hormone-like signaling molecules to oocytes and gonad sheath cells, delivered in sperm-derived vesicles, in addition to MSP performing cytoskeletal functions [66],[67]. Additionally, components of the seminal fluid that derive from cells of the male somatic gonad play important roles in reproduction [28],[68]. Such seminal factors might compromise female tissue in some way that makes it more susceptible to adverse effects of heterospecific sperm, perhaps by inducing dilation of the gonadal sheath cells bordering the distal end of the spermatheca. In *C. elegans*, *gst-4* and *daf-2* mutants show defects in dilation of the spermathecal valve [69] and many proteins affect sheath contraction [59], suggesting potential pathway targets for compounds transferred by males during mating.

In *C. elegans*, oocyte-derived chemical cues guide sperm migration to the spermathecae [59]. The attractant comprises a complex mixture of F-series prostaglandins derived from poly-unsaturated fatty acid (PUFA) precursors, whose synthesis and modulation depends on a suite of fatty acid desaturases, glutathione S-transferases, cytochrome P450s, insulin-like signaling, and communication between the somatic gonad and germ cells [69]–[72]. In *C. elegans* mutants of these genes, conspecific sperm fail to localize efficiently to the spermathecae, instead occurring more readily in the uterus [69],[70]. Heterospecific sperm migrate to the spermathecae efficiently in *C. elegans* and *C. briggsae* hermaphrodite reproductive tracts (Figure S6A–S6D) [41], indicating conservation of the sperm attractants and their detection between species. The high density of aggressive heterospecific sperm in their spermathecae then sets the stage for invasion of sperm into ectopic locations. However, sperm localization patterns for *C. tropicalis* suggests that its oocytes might secrete a sperm cue that is only weakly attractive, or a chemical mixture that attracts sperm efficiently only for some species (Figure S6B). Consequently, heterospecific sperm occur with lower density in the spermathecae, with correspondingly reduced likelihood of invasion into the distal gonad (Figure S6B). This hypothesis could underlie the limited adverse effects of heterospecific mating on *C. tropicalis* hermaphrodites. It might even implicate the weak sperm attractants as an evolutionary response through gametic reinforcement [20] if negative effects of inter-species mating in sympatry occurred in *C. tropicalis*' past [38]. Finally, it is conceivable that differences among species in sperm attraction to distinct prostaglandins, or other compounds, coupled with secretion of them by more distally developing oocytes could encourage ectopic sperm migration.

The mechanism by which sperm migrate ectopically past the spermathecal boundary remains a key unsolved problem. Moreover, the breaching of the ovary basement membrane and migration of sperm into the body cavity through amoeboid movement, bearing a striking resemblance to features of metastasis in cancer [73]–[75], motivates further investigation of the molecular basis of tissue integrity and resistance to cellular invasion [76].

## Materials and Methods

### Maintenance

Animals were maintained according to standard *C. elegans* procedures [77], with the exception of increased agar concentration in NGM plates to 2.2% in order to discourage animals from burrowing underneath the surface of the plate. Cultures were maintained at 20°C and 25°C. See Text S1 for strains of each species used for experiments: *C. afra* (sp. 7), *C. brenneri*, *C. briggsae*, *C. elegans*, *C. latens* (sp. 23), *C. nigoni* (sp. 9), *C. portoensis* (sp. 6), *C. remanei*, *C. tropicalis* (sp. 11), *C. wallacei* (sp. 16), *C.* sp. 5 [44].

### Quantification of Reduced Reproductive Output

Crosses consisted of placing one hermaphrodite at the fourth larval stage (L4, penultimate stage of development) with six heterospecific males overnight (18–24 hours) on a 35 mm diameter Petri dish with a 10 mm diameter bacteria spot (*E. coli* OP50). Hermaphrodites (Figure 1A–1F) that successfully mated (presence of a copulatory plug) were transferred daily and we measured reproductive output as the yield of viable adult progeny from two days of egg laying following the final mating event (representing >90% of lifetime brood size). Control hermaphrodites were individuals allowed to produce self-progeny.

For matings involving females (Figure 2A and 2B), we first mated them to conspecific males overnight and the subsequent day we mated treatment females to heterospecific males. In all cases except one (*C. briggsae*×*C. nigoni*), matings are incapable of yielding viable hybrid progeny (few hybrids are produced by *C. briggsae*×*C. nigoni* [40],[78]). Therefore, reproductive output measures the number of conspecific progeny of females (or, equivalently, self-progeny of hermaphrodites).

The single mating treatment (Figure 1D) consisted of placing ten young adult hermaphrodites (*C. briggsae*) with 40 young adult males (*C. nigoni*) on a 35 mm Petri dish with a 10 mm diameter bacteria spot. After an hour, mated hermaphrodites were isolated to individual 35 mm diameter Petri dishes, transferred daily, and allowed to lay eggs in order to measure progeny production.

### Survival Measurements

In situations when both female and hermaphrodites are used, they will be referred to as XX animals as they both have two X chromosomes. Seven L4 XX animals, depending on the species, were placed with ten heterospecific or conspecific males per plate and left overnight. The next day, XX animals were assayed for mortality by being touched on the head with an eyebrow hair glued to a toothpick. If the animal performed a backwards locomotive response to the touch, it was scored as alive. If it did not, it was scored as dead. This was performed every day for at least seven days. Every two days, XX animals and males were transferred to new plates in order to prevent the confusion of progeny with parents. Additionally, in these assays, XX animals were kept under continuous mating conditions: when males died or crawled off the plate, they were replaced with new males. XX animals that crawled off the plate were excluded from the lifespan measurements.

### DNA Staining and Vital Staining of Sperm

The nuclei of animals were visualized using Hoechst 33258 staining. Seven XX animals were mated with ten heterospecific or conspecific males per plate for 1–3 days, and then XX animals were fixed in 100% methanol overnight at 4°C. The animals were then washed three times in M9 buffer and incubated in 1 µg/ml Hoechst in M9 buffer for 5 minutes, followed by mounting for fluorescent microscopy and imaging.

Male sperm were fluorescently labeled *in vivo* with MitoTracker Red CMXRos (Invitrogen) [71]. Males were incubated in 1 mM dye for 2–3 hours, and then left on a plate to recover overnight. Subsequently, these males were mated with virgin young adult XX animals for 1–4 hours (matings with *C. elegans* males were allowed to run overnight). Virginity was assured by isolating XX L4 animals from males before reaching adulthood. Mated XX animals were then mounted on 10% agarose pads [79] or 2% agarose pads and immobilized with 50 mM sodium azide for differential interference contrast (DIC) and fluorescence imaging. Automated time-lapse photography (1–10 frames per second) was performed with the Open Lab software package and a Zeiss Axioskop 2 equipped with DIC and fluorescence microscopy.

### *C. nigoni-fog-3(RNAi)* and Scoring Germ Line Feminization Phenotypes

A 929 base pair fragment including coding sequence homologous to *fog-3* was PCR amplified from *C. nigoni* genomic DNA using primers flanked with 5′ T7 promoters. The reaction was gel purified using the QIAquick kit (Qiagen), and the resultant template was then used for *in vitro* transcription using the MAXIscript kit (Ambion) to make dsRNA. The dsRNA was recovered using phenol-chloroform extraction and isopropanol precipitation, and the dsRNA was then introduced into the animals *via* maternal microinjection. The male progeny of injected animals were scored for the feminization of germline (Fog) phenotype using DIC microscopy via standard methods [77]. The worms were mounted on 2% agarose pads and immobilized with 50 mM sodium azide. Only males with clearly defined oocytes and no observable sperm were used for sterilization and lethality experiments. Fog males were allowed to recover for 30 minutes on a plate in a drop of M9 buffer. These males were capable of performing the mating behaviour and of depositing copulatory plugs (and presumably other seminal fluids). These males were then assayed for their ability to sterilize and prematurely kill *C. briggsae* hermaphrodites. These males were then used for experiments as described above. Control wild-type males were mounted, immobilized, and allowed to recover for the same amount of time in order to remove these as confounding factors.

### Assortative Mating

We focused our assortative mating assays on *C. nigoni* males, as their aggressive sperm results in sterility and increased mortality (Figure 1). We expect males to mate indiscriminately [41],[43]; therefore, XX animal behaviours (preference or avoidance) should account for the majority of mating biases observed.

Assortative mating assays consisted of placing ten virgin *C. nigoni* males with ten virgin conspecific and/or heterospecific mating partners on a 35 mm diameter Petri dish. The three treatments involved presenting males to (i) ten conspecific, (ii) ten heterospecific (*C. remanei*, *C. elegans*, or *C. briggsae*), or (iii) a mixture of five conspecific and five heterospecific mating partners. See Text S1 for results of (i) conspecific and (ii) heterospecific treatment. Control assays consisted of males (*C. remanei*, *C. elegans*, or *C. briggsae*) following the same treatments as above with *C. nigoni* females as the heterospecific species.

We recorded successful mating by the presence of a copulatory plug deposited by a male onto an XX animal's vulva. We also recorded whether any XX animals left the 3 mm diameter (5 µl) bacterial spot mating area, which, we reasoned, was effective in avoiding copulation. We limited the mating period to 10 minutes to ensure males only mated once (male∶female ratio >1 was used to more easily observe successful copulations with inefficient males of androdioecious species). This 10 minute mating period was determined by preliminary experiments with a male placed with multiple conspecific females. In order to visually distinguish the two female/hermaphrodite species from one another, strains with pharyngially expressed GFP (*C. briggsae* PS9391) or RFP (*C. nigoni* VX0092) markers were used, which we presume exerts no direct effect on mate choice. See Text S1 for observed mating frequencies.

### Statistical Analyses

All statistical analyses were performed using IBM SPSS Statistics v.20, unless otherwise noted. We conducted non-parametric tests for measures of reproductive output, owing to non-normal distributions and heterogeneous variances. To assess the effect of heterospecific matings on reproductive output (i.e., extent of sterilization), we compared the control (selfing for hermaphrodites and conspecific matings for females) to each treatment (heterospecific mating) using Mann-Whitney U tests with Bonferroni correction for multiple testing. We used Kaplan-Meier survival analysis to test for an effect of mating on survival of females or hermaphrodites. The survival analyses were performed with the OASIS online application [80] and SPSS.

In experiments that explored assortative mating with a mixed species treatment (five conspecific and five heterospecific mating partners; Figure 6), an index of mating bias was calculated as the difference between the number of mated *C. nigoni* females and the number of mated individuals of the other maternal species present in the arena, divided by the number of *C. nigoni* females present in the arena (five). Positive values indicate a mating bias towards *C. nigoni* females over the female (or hermaphrodite) species that they were paired with, negative values indicate the reciprocal, and a value of zero indicates no mating bias (a lack of preference or avoidance). Negative values were not observed in our experiments. We then tested for a significant difference from zero with two-tailed one sample *t*-tests.

## Supporting Information

Figure S1 ***C. nigoni*** **females mate multiply.** (A) DIC image of a *C. nigoni* adult hermaphrodite after a two hour mating period with conspecific males labeled with vital dyes (red or green). Diakinesis stage oocytes (do) are seen distal to the uterus, but not beyond the bend in the reflexed gonad (asterisk). (B, C) Sperm (sp) from males stained with red and green are localized in the spermatheca. Also denoted is the vulva (v), and embryos localized to the uterus (emb). (D) A merged image of (A–C). (C, D) Auto fluorescence of the gut is visible. Each panel is a mosaic assembled from multiple overlapping images; all scale bars are 100 microns. See Text S1 for quantification of multiple mating experiments.
(TIF)

Figure S2 **Inter-species mating harm is neither prevented nor substantially rescued by conspecific mating.** *C. briggsae* hermaphrodites were left unmated (selfing only), treated to one mating period with one set of males (Con only: conspecific *C. briggsae* or Het only: heterospecific *C. nigoni*), or treated to two mating periods with two sets of males (Het-Het, Het-Con, Con-Het, Con-Con). A conspecific mating taking place one day after a heterospecific mating does not strongly rescue *C. briggsae* progeny production (Het-Het versus Het-Con: Mann-Whitney U = 126.5, *p* = 0.043: not significant after multiple tests corrections). Additionally, a conspecific mating does not prevent sterilization from a subsequent heterospecific mating (Con-Con versus Con-Het: U = 5.5, *p*≤0.001). Finally, *C. briggsae* hermaphrodites mated with heterospecific males, regardless of order, experience the same decrease in reproductive output (Het-Con versus Con-Het: U = 208.0, *p* = 0.599). Dotted line indicates the median of the selfing control for reference; samples sizes are in parentheses. Boxplot whiskers indicate 1.5× (interquartile range). For all mating treatments reproductive output is quantified by adult progeny produced two days following the second mating period. Multiple Mann-Whitney U tests were conducted and Bonferroni correction for multiple tests were applied (corrected α = 0.0125). Asterisk indicate statistical significance and (ns) indicate non-significance following Bonferroni correction.
(EPS)

Figure S3 **No correlation between severity of harm by heterospecific males and phylogenetic distance or disparity in sperm size.** We constructed an ordinal scale of severity of harm to females (or hermaphrodites) as: 1 = no sterilization and/or ectopic sperm; 2 = weak progeny reduction and/or no ectopic sperm; 3 = moderate progeny reduction and/or some ectopic sperm detected; 4 = strong sterilization and/or ectopic sperm present; 5 = near complete sterilization and/or extensive ectopic sperm. (A) We plotted the metric of severity of harm as a function of discretized evolutionary distance of hermaphrodites to heterospecific males mates, on the basis of the phylogenetic topology among species (see Figure 1). We observed no significant relation between phylogenetic distance and severity of harm, either for all hermaphrodite species pooled (Spearman's ρ = −0.15, *p* = 0.52) or considered separately (all distances have identical harm index for *C. briggsae*; *C. elegans* ρ = −0.54, *p* = 0.28, *C. tropicalis* ρ = −0.17, *p* = 0.74). Although this analysis is crude and is based on only a modest number of species comparisons, a more sophisticated analysis of phylogenetic contrasts is undermined by the topology of phylogenetic relationships among the species included in our study. (B) On the basis of sperm size values for the subset of species available in [48], we computed the difference in sperm cross-sectional area between conspecific and heterospecific males. In the case of matings to hermaphrodites, size of hermaphrodite sperm was used in calculations. Spearman rank correlation between these phylogenetically uncorrected metrics was not significant for matings to heterospecific males from dioecious species (*p* = 0.32). In the case of matings to androdioecious males, we detected a significant association (*p* = 0.012) owing to the weak sensitivity of hermaphrodites and no sensitivity of females to heterospecific androdioecious males.
(EPS)

Figure S4 **Patterns of maternal survival in different** ***Caenorhabditis*** **crosses.** Maternal survival in conspecific and heterospecific crosses of (A) *C. tropicalis* (hermaphrodite/male) with *C. wallacei* (female/male); (B) *C.* sp. 5 (female/male) with *C. nigoni* (female/male); and (C). *C. remanei* (female/male) with *C. nigoni* (female/male). (A) *C. wallacei* maternal survival was statistically greater in the presence of *C. tropicalis* males (Kaplan-Meier log-rank test: χ^2^ = 49.57, df = 1, *p*≤0.001). However, *C. tropicalis* maternal survival was not affected by *C. wallacei* males (χ^2^ = 0.45, df = 1, *p* = 0.503). (B) The presence of heterospecific males had no statistically significant effect on survival of *C.* sp. 5 females (χ^2^ = 0.002, df = 1, *p* = 0.961) or *C. nigoni* females (χ^2^ = 0.16, df = 1, *p* = 0.688). (C) *C. remanei* maternal lifespan over eight days was significantly reduced in the presence of *C. nigoni* males (χ^2^ = 8.81, df = 1, *p* = 0.003). *C. nigoni* maternal survival was not decreased by *C. remanei* males (χ^2^ = 1.65, df = 1, *p* = 0.199). Sample sizes are in parentheses, asterisks indicate significant difference in survival (*p*≤0.05), and *ns* indicate non-significant difference in survival. In all panels, dashed lines represent conspecific crosses and solid lines represent heterospecific crosses, while colours correspond to the maternal species.
(EPS)

Figure S5 ***C. nigoni*** **sperm can ectopically localize and fertilize oocytes in** ***C. elegans*** **hermaphrodites.** (A–D) Two *C. elegans* hermaphrodites mated for 2–6 hours with vitally stained *C. nigoni* males. Panels display images under DIC (A, C) and fluorescence microscopy (B, D). Indicated is the presence of ectopically localized *C. nigoni* sperm (esp). (E) A different focal plane of the animal in panels (C, D) reveals the presence of an ectopic embryo (ece) distal to the spermatheca. Also denoted are sperm (sp), the vulva (v), and a properly localized embryo (emb). The bend of the gonad is noted by an asterisk. All scale bars are 100 microns.
(TIF)

Figure S6 **Mislocalization of sperm in different** ***Caenorhabditis*** **crosses.** The percent of mated females/hermaphrodites observed to contain fluorescently labeled male sperm that localize outside of the spermatheca and uterus (ectopic, purple), in the uterus (orange), and/or in the spermathecae (blue). (A, B) Species names in black boxes below the x-axis indicate dioecious species; white boxes indicate androdioecious species. (C) *C. elegans fog-2* “females” doubly mated to vitally stained males (red or green) show that the smaller *C. elegans* male sperm are displaced from the spermatheca by larger *C. nigoni* sperm. (D) The percent of observed *C. briggsae* hermaphrodites with ectopic *C. nigoni* male sperm increases with time since mating. The dotted horizontal line denotes a 50% reference line; sample sizes are in parentheses.
(EPS)

Table S1 **Statistical results of multiple Mann-Whitney U tests corresponding to** **Figure 1A–1C** **.**
(DOCX)

Movie S1 **Male sperm localization in mated** ***C. briggsae*** **hermaphrodites.** (A) Labeled *C. briggsae* male sperm localize to the spermatheca and uterus of a *C. briggsae* hermaphrodite. Time-lapse video of a *C. briggsae* hermaphrodite that has been mated with a fluorescently labeled *C. briggsae* male. (B) Labeled *C. nigoni* male sperm ectopically migrate in a *C. briggsae* hermaphrodite. Time-lapse video of a *C. briggsae* hermaphrodite that has been mated with a fluorescently labeled *C. nigoni* male. Numerous sperm are located outside of the hermaphrodite's gonad. The spermatheca and uterus have been outlined in yellow, whereas the proximal and distal gonad have been labeled in white. Scale bar represents 100 microns. Images were taken every 10 seconds and the video is sped up 10×.
(AVI)

Movie S2 **Ectopic migration of an individual** ***C. nigoni*** **sperm in a** ***C. briggsae*** **hermaphrodite.** Differential interference contrast time-lapse video of a *C. briggsae* hermaphrodite that has been mated with a *C. nigoni* male. Three ectopic sperm are visible (indicated by the white arrowheads in the opening frames). One ectopic sperm is observed crawling transversely around the distal gonad (lone arrowhead in subsequent frames). Each frame step represents one second.
(MP4)

Text S1 **Supplementary methods and results.** Strains used in each experiment. Results of mating frequencies observed from assortative mating assays. Additional experiments examining sperm competition within species, effects of conspecific matings to heterospecifically mated hermaphrodites, and sperm localization in different species of *Caenorhabditis*.
(DOC)

## Abbreviations

df: degree of freedom
DIC: differential interference contrast
Fog: feminization of germline
XX animals: females and hermaphrodites
