# Supplementary figures and images for: Intense Sperm-Mediated Sexual Conflict Promotes Reproductive Isolation in Caenorhabditis Nematodes
Source: PLoS Biol. 2014 Jul 29;12(7):e1001915. doi: 10.1371/journal.pbio.1001915 (PMC4114750; doi:10.1371/journal.pbio.1001915)

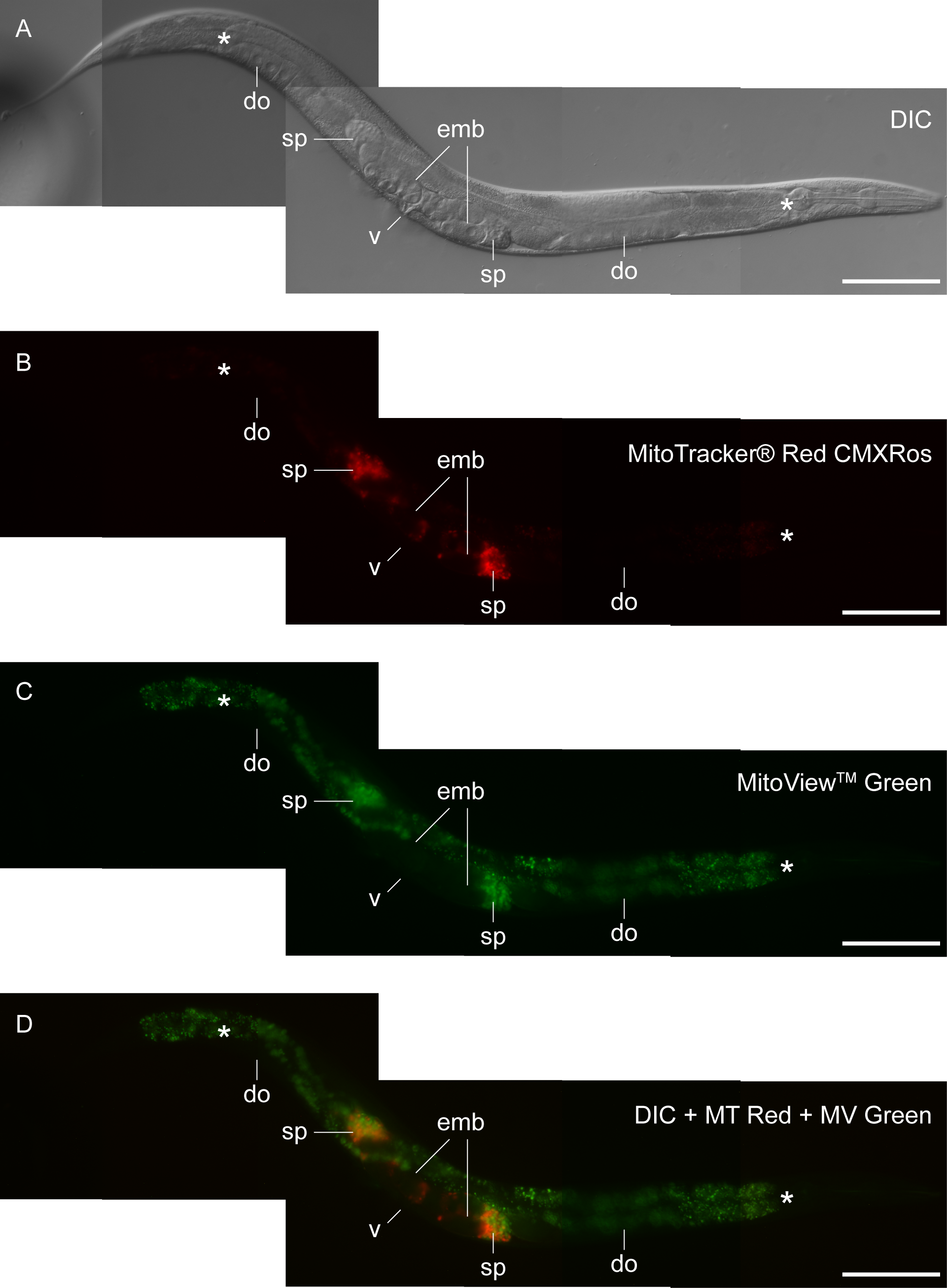

Supplement: Figure S1 — C. nigoni females mate multiply. (A) DIC image of a C. nigoni adult hermaphrodite after a two hour mating period with conspecific males labeled with vital dyes (red or green). Diakinesis stage oocytes (do) are seen distal to the uterus, but not beyond the bend in the reflexed gonad (asterisk). (B, C) Sperm (sp) from males stained with red and green are localized in the spermatheca. Also denoted is the vulva (v), and embryos localized to the uterus (emb). (D) A merged image of (A–C). (C, D) Auto fluorescence of the gut is visible. Each panel is a mosaic assembled from multiple overlapping images; all scale bars are 100 microns. See Text S1 for quantification of multiple mating experiments. (TIF) [file pbio.1001915.s001.tif]

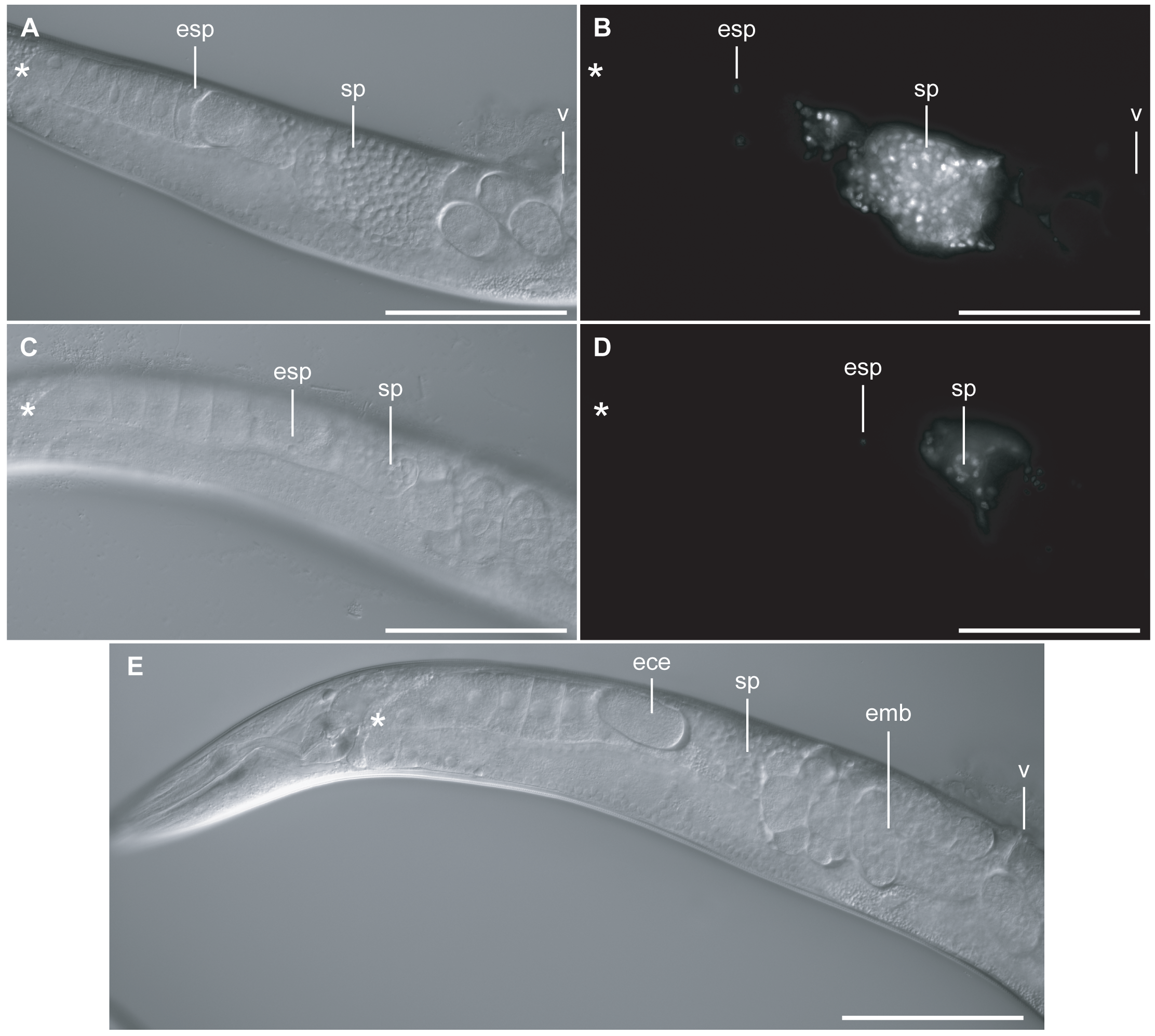

Supplement: Figure S5 — C. nigoni sperm can ectopically localize and fertilize oocytes in C. elegans hermaphrodites. (A–D) Two C. elegans hermaphrodites mated for 2–6 hours with vitally stained C. nigoni males. Panels display images under DIC (A, C) and fluorescence microscopy (B, D). Indicated is the presence of ectopically localized C. nigoni sperm (esp). (E) A different focal plane of the animal in panels (C, D) reveals the presence of an ectopic embryo (ece) distal to the spermatheca. Also denoted are sperm (sp), the vulva (v), and a properly localized embryo (emb). The bend of the gonad is noted by an asterisk. All scale bars are 100 microns. (TIF) [file pbio.1001915.s005.tif]
