# Supplementary material for: Intense Sperm-Mediated Sexual Conflict Promotes Reproductive Isolation in Caenorhabditis Nematodes
Source: PLoS Biol. 2014 Jul 29;12(7):e1001915. doi: 10.1371/journal.pbio.1001915 (PMC4114750; doi:10.1371/journal.pbio.1001915)
Supplement: Table S1 — Statistical results of multiple Mann-Whitney U tests corresponding to Figure 1A–1C . (DOCX) [file pbio.1001915.s007.docx]

**Table S1.** **Statistical results of multiple Mann-Whitney U tests corresponding to Figure 1A-C**. Hermaphrodites of each selfing species were mated to males of outcrossing species and the number of viable progeny produced (two days post mating treatment) was compared to progeny of selfing or ‘no mating’ hermaphrodites. **(A)** *C. briggsae* hermaphrodites selfing (n=61) compared to heterospecific crosses to seven paternal species. Bonferroni correction for multiple tests was applied; corrected α=0.007. **(B)** *C. elegans* hermaphrodites selfing (n=58) compared to heterospecific crosses to seven paternal species. Bonferroni correction for multiple tests was applied; corrected α=0.007. **(B)** *C. tropicalis* hermaphrodites selfing (n=32) compared to heterospecific crosses to seven paternal species; no successful matings were observed with *C. portoensis* males. Bonferroni correction for multiple tests was applied; corrected α=0.008. Asterisks indicate statistically significant relationships after multiple tests corrections.

**A**

| **Maternal** | **Paternal** | **n** | **U** | **P** |
| --- | --- | --- | --- | --- |
| *C. briggsae* | *C. nigoni* | 29 | 42.0 | **≤0.001 *** |
|  | *C.* sp. 5 | 31 | 16.0 | **≤0.001 *** |
|  | *C. remanei* | 26 | 29.0 | **≤0.001 *** |
|  | *C. latens* | 22 | 0.0 | **≤0.001 *** |
|  | *C. brenneri* | 21 | 0.0 | **≤0.001 *** |
|  | *C. afra* | 33 | 0.0 | **≤0.001 *** |
|  | *C. portoensis* | 28 | 99.5 | **≤0.001 *** |

**B**

| **Maternal** | **Paternal** | **n** | **U** | **P** |
| --- | --- | --- | --- | --- |
| *C. elegans* | *C. nigoni* | 26 | 30.0 | **≤0.001 *** |
|  | *C.* sp. 5 | 36 | 87.0 | **≤0.001 *** |
|  | *C. remanei* | 30 | 37.0 | **≤0.001 *** |
|  | *C. latens* | 32 | 0.0 | **≤0.001 *** |
|  | *C. brenneri* | 25 | 212.5 | **≤0.001 *** |
|  | *C. afra* | 24 | 65.5 | **≤0.001 *** |
|  | *C. portoensis* | 35 | 221.0 | **≤0.001 *** |

**C**

| **Maternal** | **Paternal** | **n** | **U** | **P** |
| --- | --- | --- | --- | --- |
| *C. tropicalis* | *C. nigoni* | 32 | 433.0 | **≤0.001 *** |
|  | *C.* sp. 5 | 25 | 364.0 | **0.002 *** |
|  | *C. remanei* | 24 | 431.5 | 0.031 |
|  | *C. latens* | 26 | 514.0 | 0.086 |
|  | *C. brenneri* | 20 | 31.5 | **≤0.001 *** |
|  | *C. afra* | 31 | 301.0 | **≤0.001 *** |
|  | *C. portoensis* | 68 | Did not mate | |
