# Supplementary material for: Intense Sperm-Mediated Sexual Conflict Promotes Reproductive Isolation in Caenorhabditis Nematodes
Source: PLoS Biol. 2014 Jul 29;12(7):e1001915. doi: 10.1371/journal.pbio.1001915 (PMC4114750; doi:10.1371/journal.pbio.1001915)
Supplement: Text S1 — Supplementary methods and results. Strains used in each experiment. Results of mating frequencies observed from assortative mating assays. Additional experiments examining sperm competition within species, effects of conspecific matings to heterospecifically mated hermaphrodites, and sperm localization in different species of Caenorhabditis. (DOC) [file pbio.1001915.s010.doc]

**Text S1. Supplementary methods and results.**

**Strains used in each experiment**

**Quantification of reduced reproductive output** (Fig. 1, 2, S2) **-** Strains of androdioecious species used: *C. elegans* CB4856, *C. briggsae* AF16, and *C. tropicalis* JU1373. Strains of dioecious species used: *C. nigoni* JU1421, *C.* sp. 5 JU800, *C. remanei* PB272, *C. latens* VX0088, *C. brenneri* CB5161, *C. afra* JU1199, and *C. portoensis* EG4788.

**Survival measurements** (Fig. 1, 2, S4) -*C. briggsae* AF16, *C*. *nigoni* JU1325, *C. remanei* EM464, *C. elegans* *him-5(e1490)* DR461, *C. tropicalis* JU1373, and *C. wallacei* JU1873,

**DNA staining and vital staining of sperm** (Fig. 3, 4, S5, S6A, Movie S1) -*C. briggsae* AF16, *C. tropicalis* JU1373, *C. elegans* *him-5(e1490)* DR461, *C*. *nigoni* JU1325, and *C. remanei* EM464.

***C. nigoni* -*fog-3(RNAi)* and scoring germ line feminization phenotypes** (Fig. 5) **-** *C. briggsae* AF16, *C*. *nigoni* JU1325, *C. remanei* EM464, and *C. elegans* *him-5(e1490)* DR461.

**Assortative Mating** (Fig. 6) - *C. briggsae* AF16 and PS9391, *C. elegans* CB4856, *C. remanei* PB272, and *C. nigoni* JU1421 and VX0092.

**Sperm competition within species** (Fig. S1) -*C. nigoni* JU1421, and *C. remanei* PB272.

**Mislocalization of sperm in different *Caenorhabditis* crosses** (Fig. S6B-D) **-** *C. briggsae* AF16, *C. tropicalis* JU1373, *C. elegans* CB4856, *C. nigoni* JU1325, *C. remanei* EM464, and *C. brenneri* CB5161 (Fig. S6B, S6D).

*C. elegans fog-2* “females” JK574, *C. elegans* males CB4856, and *C. nigoni* males JU1325 (Fig. S6C).

**Assortative Mating:** *Mating frequencies*

There are three treatments for the assortative mating assay: 10 virgin *C.* *nigoni* males were presented to i) 10 conspecific females, ii) 10 heterospecific females/hermaphrodites (*C. remanei*, *C. elegans*, or *C. briggsae*), or iii) a mixture of five conspecific and five heterospecific mating partners. Control assays consisted of males (*C. remanei*, *C. elegans*, or *C. briggsae*) following the same treatments as above with *C. nigoni* females as the heterospecific species. Here we present results from treatment i and ii for both treatment and control assays; see main text for results of treatment iii.

Results of i and ii provide estimates on mating frequencies of females and hermaphrodites to conspecific and heterospecific males. Sample sizes of assortative mating trials were 25 for all treatments and controls for the species pair *C. briggsae* x *C. nigoni*, and species pair *C. remanei* x *C. nigoni*. For treatment and control trials of species pair *C. elegans* x *C. nigoni*, the sample sizes were 20. To test for differences in mating frequencies of the maternal species to each male species, we compared the proportion of plugged individuals after 10 minutes for each species pair using a two sample t-test.

*C. nigoni* females mated more frequently to *C. nigoni* males than *C. remanei* females (t=-2.518, df=50, P=0.015), *C. elegans* hermaphrodites (t=-21.387, df=21.959, P≤0.001; corrected for unequal variances), and *C.* *briggsae* hermaphrodites (t=-10.623, df=40.130, P≤0.001; corrected for unequal variances). There was no difference in mating frequency of *C. nigoni* females and conspecific maternal partners to *C. remanei* males (t=-1.854, df=43.875, P=0.070), *C. elegans* males (t=0.461, df=38, P=0.648), and *C. briggsae* males (t=0.576, df=48, P=0.567).

**Measuring sperm competition within species**

In order to assess the frequency of multiple matings in conspecific groups of *C. nigoni* and *C. remanei* (see Fig. S1) we placed 10 virgin females with five red and five green fluorescently stained conspecific males (see below for staining methods). After two hours, all females from both species (n=11) were mated (indicated by the presence of a mating plug). Females were examined for the presence of red, green or both fluorescent colours of sperm in the reproductive tract.

We observed that 22% ± 3.1% (mean ± 1SE) *C. nigoni* females (n=11) and 12% ± 3.1% *C. remanei* females (n=11) had both red and green sperm in their reproductive tract. All of the remaining females had sperm of only one colour: 37% ± 4.0% (red) and 41% ± 4.9% (green) of *C. nigoni* females; 62% ± 5.1% (red) and 26% ± 3.2% (green) of *C. remanei* females. We estimate the probability of *C.* *nigoni* and *C. remanei* females having mated with more than one male in the two hour timeframe to be 66% and 36%, respectively.

Males were stained overnight on an agar plate (35 mm diameter Petri dish) with mitochondrial specific fluorescent dyes: MitoTracker® Red CMXRos (Molecular Probes) and MitoView^TM^ Green (Biotium). Three 5 μL drops of each stain were applied to a 5 μL bacterial spot on each 3 cm NGM plate. MitoTracker® Red CMXRos was diluted in DMSO to a concentration of 1 mM and was further diluted with M9 to a final concentration of 100 μM. MitoView^TM^ Green stock solution (200 μM) was applied directly to the bacterial spot. Approximately 100 male worms were added to each plate and incubated in the dark overnight (18-24 hours).

**Quantification of reduced reproductive output: Effects of conspecific matings to heterospecifically mated hermaphrodites**

We examine the effects of conspecific matings prior to or following heterospecific matings to *C. briggsae* hermaphrodites (Fig. S2). Hermaphrodites were left unmated (selfing), treated to one mating period (18-24 hours) with one set of six males (*C. briggsae* or *C. nigoni*), or treated to two mating periods with two sets of males. For the double mating period crosses there were two treatments: one mating period with *C. briggsae* males followed by a second mating period with *C.* *nigoni* males (Con – Het), and the reciprocal (Het – Con). We also controlled for the effects of multiple mating periods by mating *C*. *briggsae* hermaphrodites to two rounds of males of the same species (Con – Con or Het – Het). Reproductive output was measured by the number of progeny laid two days following the second mating period for all treatments (regardless if a second mating occurred). Mann-Whitney U tests were conducted to test for differences in viable progeny produced. Bonferroni correction for multiple tests was applied with a corrected α of 0.0125.

Similar to previous results, the reproductive output of *C. briggsae* hermaphrodites unmated (selfing) differed from when mated to a single set of conspecific males (Mann-Whitney U=21.0, P≤0.001) and heterospecific males (U=0, P≤0.001). Additionally, *C. briggsae* hermaphrodites mated to conspecific males produce significantly more progeny than when mated to heterospecific *C. nigoni* males (U=0, P≤0.001). This result remained when hermaphrodites were mated two days in a row (Het – Het vs. Con – Con: U=1.0, P≤0.001). We found no effect of multiple mating periods on reproductive output: single versus double mating with the same male species (Het only vs. Het – Het: U=131.5, P=0.222; Con only vs. Con – Con: U=171.5, P=0.102). A prior conspecific mating result in more progeny compared to two consecutive matings with heterospecific males (Con – Het vs. Het – Het: U121.0, P=0.007), while a prior heterospecific mating results in fewer progeny compared to two consecutive matings with conspecific males (Het – Con vs. Con – Con: U=31.0, P≤0.001).

**Mislocalization of sperm in different *Caenorhabditis* crosses**

Males were incubated in 1 mM dye for 2-3 hours, and then left on a plate to recover overnight. Subsequently, these males were mated with virgin young adult XX animals.

Hermaphrodites were placed with stained males (MitoTracker® Red CMXRos) in a 3 cm NGM plate with a spot of *E. coli* at a sex ratio of 10:7 (males:hermaphrodites). After a period of 2-6 hours, mated females and hermaphrodites (presence of a mating plug) were examined. In Figure S6A, mated individuals were scored as having sperm outside of the uterus and sperm (ectopic) or not. The mated individuals were mounted on 2% agarose pads and immobilized with 50 mM sodium azide for scoring.

In Figure S6B, location of sperm was categorized as being in the uterus, either of the spermathecae, and/or ectopic (outside of the uterus and spermathecae). An individual could have sperm present in all three locations, therefore percentages do not sum to 100% (Fig. S6B). *C. elegans* males did not leave copulatory plugs on mated *C. tropicalis* hermaphrodites, therefore successful sperm transfer were verified by using a dissecting scope under fluorescent lighting. Mated hermaphrodites were examined after (mean ± SE) 3.18 ± 0.16 hours in the presence of stained males.

*Doubly mated females*

Males were stained overnight with mitochondrial specific fluorescent dyes: MitoTracker® Red CMXRos and MitoView^TM^ Green following the protocol used above in *‘Measuring sperm competition within species’*. *C. elegans fog-2* “females” were first mated to conspecific males at a sex ratio of 3:1 (males:females) and then left to mate with a second set of males stained a different colour for (mean ± SE) 3.67 ± 0.15 hours. The mated females were then mounted on 2% agarose pads and immobilized with 50 mM sodium azide for scoring location of sperm. Data shown include females with both red and green sperm present (Fig. S6C).

Females mated with two sets of conspecific males had sperm of both colours localized in both the uterus and spermatheca (Fig. S6Ci). However, when females are mated first to conspecific males and then heterospecific *C. nigoni* males, the sperm from both male species are not equally distributed (Fig. S6Cii-iii). The larger heterospecific male sperm displaces the conspecific male sperm from the spermatheca (the site of fertilization). The majority of conspecific male sperm is found in the uterus, where the sperm must crawl back to the spermatheca or risk being pushed out with fertilized eggs.

*Time dependent*

*C. nigoni* males were incubated in 1 mM dye for 2-3 hours, and then left on a plate to recover overnight. Subsequently, they were placed with *C. briggsae* hermaphrodites at a sex ratio of 10:7 (males:hermaphrodites). After 30 minutes, plugged hermaphrodites were transferred to a new plate and kept in the dark as the fluorescent stain is light sensitive. Mated hermaphrodites were then mounted on 2% agarose pads and immobilized with 50 mM sodium azide for scoring location of sperm at either 1, 3, 6, 9, or 12 hours since mating.

Only hermaphrodites with fluorescent sperm present were scored. Sperm location was categorized as being in the uterus, either of the spermathecae, in the distal and/or proximal gonad, and/or regions outside of the reproductive tract. We combine the last two categories into a single category (ectopic). This was done to account for any variation in the brightness of the fluorescent stain overtime. An individual could have sperm present in all three locations, therefore percentages do not sum to 100%

One hour post mating, 32.4% of the hermaphrodites observed had *C. nigoni* sperm localized ectopically (n=34; Fig. S6D). The percentage of hermaphrodites with ectopic sperm increased to 51.4% (n=37) three hours post mating. After six hours, at all the time points examined, over 90% of *C. briggsae* hermaphrodites had sperm located outside of the uterus and spermatheca (6 hours: 97.1%, n=35; 9 hours: 90.9%, n=33; 12 hours: 100%, n= 21; Fig. S6D).
